# Daily Dosing for Bedaquiline in Patients with Tuberculosis

**DOI:** 10.1128/AAC.00463-19

**Published:** 2019-10-22

**Authors:** David H. Salinger, Jerry R. Nedelman, Carl Mendel, Melvin Spigelman, David J. Hermann

**Affiliations:** a Certara, Inc., under contract with the Bill and Melinda Gates Foundation, Seattle, Washington, USA; b TB Alliance, New York, New York, USA; c Bill and Melinda Gates Foundation, Seattle, Washington, USA

**Keywords:** *Mycobacterium tuberculosis*, antimicrobial agents, multidrug resistance, tuberculosis

## Abstract

The bedaquiline regimen for the treatment of multidrug-resistant tuberculosis (MDR-TB) in adults is a loading dose of 400 mg QD for 2 weeks followed by 200 mg thrice weekly (TIW) for 22 weeks. Most TB antibiotics administered with bedaquiline are given QD. Using pharmacokinetic simulations, we explored alternative QD bedaquiline regimens and determined that 200 mg QD for 8 weeks followed by 100 mg QD provides comparable exposures to the approved regimen. This simpler regimen is under clinical evaluation.

## Abstract (precis)

The bedaquiline regimen for the treatment of multidrug-resistant tuberculosis (MDR-TB) in adults is a loading dose of 400 mg QD for 2 weeks followed by 200 mg thrice weekly (TIW) for 22 weeks. Most TB antibiotics administered with bedaquiline are given QD.

## TEXT

Tuberculosis (TB) is an infectious disease caused by the Mycobacterium tuberculosis bacterium. TB primarily affects the lungs but can spread to other parts of the body. In 2017, there were an estimated 10 million new cases of TB and an estimated 1.6 million TB-related deaths worldwide (1).

Bedaquiline, as part of combination therapy, received accelerated approval in the United States for treatment of pulmonary multidrug-resistant TB (MDR-TB) in adults when an effective regimen cannot otherwise be provided (2). The labeled regimen includes a 2-week loading dose of 400 mg once daily (QD) followed by thrice weekly (TIW) doses of 200 mg for 22 weeks. For treatment of TB, bedaquiline is combined with other antibiotics, which are typically administered QD.

The work described herein was undertaken to support the use of uniform QD bedaquiline dosing, which would simplify use when administered with the other components of a regimen. A report on this work was submitted to the FDA as part of the justification of the new QD regimen being tested currently in two clinical trials (3, 4). Bedaquiline plasma exposures for several QD bedaquiline regimens are compared to the currently labeled regimen using pharmacokinetic simulations based on published population pharmacokinetic models (5, 6). A QD regimen is proposed that is predicted to provide similar drug exposure as the labeled regimen.

### Pharmacokinetic model.

A published population pharmacokinetic (PK) model (McLeay et al. [5]) described the time course of bedaquiline plasma exposures in both healthy subjects and drug-sensitive (DS) and MDR-TB patients. The model was developed based on a comprehensive data set of 9 studies which included 480 individuals and 5,222 pharmacokinetic observations collected during up to 24 weeks of dosing and up to 98 weeks follow-up post-final dose. The model showed robust concordance between observed and model-predicted values across the full range of observed bedaquiline exposures and sampling times.

The model included a dual-path oral absorption component, 4-compartment disposition, and between-subject random effect terms for apparent clearance, apparent volume of distribution, the bioavailable fraction, and the relative fraction of dose to each of the two absorption pathways.

The model, including between-subject variance terms, was programmed anew in NONlinear Mixed Effects Modeling (NONMEM) version 7.3 for simulations. The NONMEM code is provided in the Supplemental Material. Graphical displays and summary tabulations were performed using R version 3.5.2.

Aspects of the model that captured key pharmacokinetic differences between subjects are as follows:

• Clearance (CL in the NONMEM code) increased 37.5% for healthy subjects or DS-TB patients compared to MDR-TB patients.
• Clearance increased 52% for black compared to nonblack race.
• Volume of the central compartment (VC in the NONMEM code) decreased 15.7% for female compared to male.
• Oral bioavailability (FTOTAL in the NONMEM code) increased 51% for healthy subjects and DS-TB patients compared to MDR-TB patients.

It should be noted that differences in bioavailability due to DS-TB compared to MDR-TB patients were attributed in the publication to “study” rather than patient type, as there were no studies with a mix of patient types. It is also possible that other factors confounded the differences attributed here to patient type. It should also be noted that DS-TB and MDR-TB effects reported for CL and F should not be interpreted in isolation, as the impact is largely offsetting (i.e., higher CL and higher F results in minor changes in CL/F).

A second published PK model in MDR-TB patients (Svensson et al. [6]) was also considered. The McLeay (5) model was originally chosen for this work because it included 7 additional studies (plus the two included in Svensson) and covered a greater variety of subjects (including DS-TB patients and healthy subjects). An advantage of the Svensson model was that it also predicts the levels of metabolite M2, which is implicated in corrected QT (QTc) prolongation (2). Pharmacokinetic simulations for parent and M2 were also undertaken using the Svensson model, and the associated figures and tables are presented in the Supplemental Material. Code for the Svensson model is available at http://repository.ddmore.foundation/model/DDMODEL00000219#Overview.

### Model qualification.

As qualification of the model and simulation programming, a visual predictive check was undertaken comparing model predictions to a test set of pharmacokinetic data collected in a clinical trial (NC-005) (7) that was not used to develop the model. The models were used as published; no parameter reestimation was performed.

Study NC-005 was a 56-day phase 2 serial-sputum-culture-conversion study in DS-TB and MDR-TB patients. It included three treatment arms, where bedaquiline was administered (as part of a combination) using either the labeled regimen to DS-TB patients (*n* = 59) or 200 mg given QD for 8 weeks to both DS-TB (*n* = 59) and MDR-TB (*n* = 60) patients (Table 1).

**TABLE 1:** Summary of study NC-005 data used in the visual predictive check^*a*^

Arm (TB population)	BDQ dose	Total no. of patients	No. of black patients (%)	Median wt (kg) (range)	Median age (yr) (range)	Median albumin (g/dl) (range)
B(loading dose)PaZ (DS-TB)	400 mg QD × 14 days, 200 mg TIW × 42 days	59	46 (78%)	55.0 (38.5–99.8)	31 (18–69)	3.3 (18.9–43.0)
B(200mg)PaZ (DS-TB)	200 mg QD × 56 days	59	48 (81%)	52.3 (37.0–83.9)	32 (19–59)	3.5 (2.27–4.30)
B(200mg)PaMZ (MDR-TB)	200 mg QD × 56 days	60	53 (88%)	51.9 (35.0–71.0)	32 (18–69)	35.0 (2.40–4.41)
Total		178	147 (83%)	53.0 (35.0–99.8)	32 (18–69)	3.50 (1.89–4.41)

a BDQ or B, bedaquiline; Pa, pretomanid; M, moxifloxacin; Z, pyrazinamide.

Simulations were conducted to mimic the population in the three arms of study NC-005 that included bedaquiline. Actual subject race, sex, TB type, and nominal dosing were used in the simulation data set. (For qualification of the Svensson model, actual subject age, race, baseline body weight and albumin levels, and nominal dosing were used in the simulation data set; see the Supplemental Material). Each subject was simulated 500 times for use in the predictive check.

**FIG 1:**
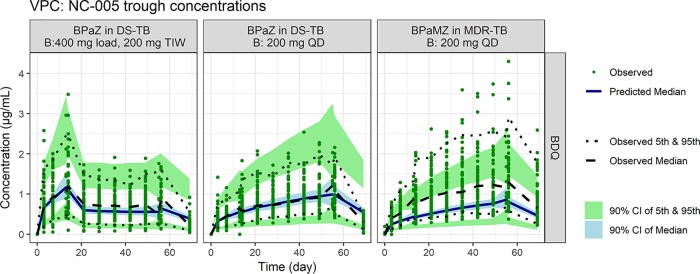
Observed and simulated bedaquiline trough concentrations over time by regimen and patient type. Visual predictive check of simulations and observed data from NC-005. Note: Median and 5th and 95th percentiles of observed bedaquiline trough concentrations and predicted 90% CI of median and 5th and 95th percentiles of predicted trough concentrations. B, bedaquiline; M, moxifloxacin; Pa, pretomanid; Z, pyrazinamide; CI, confidence interval.

**FIG 2:**
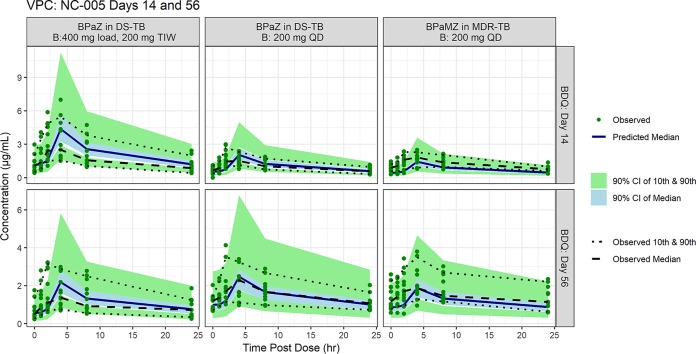
Observed and simulated bedaquiline concentrations post-final dose over time by regimen and patient type on days 14 (top row) and 56 (bottom row). Visual predictive check of simulations and observed data from NC-005. Note: Median and 10th and 90th percentiles of observed bedaquiline concentrations and predicted 90% CI of median and 10th and 90th percentiles of predicted concentrations postdose on days 14 and 56. The 80% prediction interval (10th and 90th percentiles) is used due to the small number of subjects per arm (*n* = 15) included in the substudy. B, bedaquline; M, moxifloxacin; Pa, pretomanid; Z, pyrazinamide; CI, confidence interval.

Fig. 1 and 2 depict the visual predictive checks comparing simulated versus observed distributions of bedaquiline exposures based on the McLeay model. Fig. 1 considers trough concentrations over multiple visits, while Fig. 2 considers the 24-h concentration profile immediately after dosing on days 14 and 56. Fig. S1, S2a, and S2b in the Supplemental Material present analogous results for the Svensson model.

Apart from underprediction of troughs for MDR-TB patients, the simulations were generally consistent with observations in terms of overall trends and spread, with some finer-level discrepancies, notably, overprediction of troughs on day 70 for DS-TB patients and overprediction of profile medians for the label regimen. The Svensson model demonstrated a similar general consistency with a different pattern of discrepancies in detail, most notably, greater underprediction of trough data. Nonetheless, the concordance of models and data was judged adequate for comparative simulation of alternative regimens, one of which would be selected for evaluation in two clinical trials.

### Simulation of alternative bedaquiline regimens.

The published models were then used to simulate various 6-month bedaquiline regimens, including:

• 400 mg QD for 14 days followed by 200 mg TIW (labeled regimen)
• 100 mg QD
• 200 mg QD
• 200 mg QD for 2 months followed by 100 mg QD

Each regimen of interest was simulated using the published model and assuming male DS-TB patients of black or nonblack race. Female subjects were not investigated, since the small (15.7%) sex difference in central volume (McLeay model) would not be expected to contribute substantially to relative concentration levels between the regimens under consideration. (For the Svensson model-based simulations, MDR-TB patients of black or nonblack race with body weight 53 kg, age 32 years, and baseline albumin level 3.5 g/dl were simulated; sex was not a covariate. Results are in the Supplemental Material.)

Table 2 provides summary exposure metrics for the respective regimens, including median (and 90% prediction interval of) maximum concentration of drug in serum (*C_max_*), area under the concentration time profile (AUC) over an interdose period, and cumulative AUC.

**TABLE 2:** Summary of predicted median (5th and 95th percentiles) bedaquiline exposure metrics by regimen and race based on the McLeay model^*a*^

Regimen	*C_max_* (μg/ml) (range)	*C_max_* (% of standard regimen)	Cuml. AUC at end of treatment (μg · h/ml)	Cuml. AUC (% of standard regimen)	AUC at end of treatment (μg · h/ml)	AUC (% of standard regimen)
Black patients						
Labeled regimen: 400 mg QD × 14 days, then 200 mg thrice wkly	4.07 (1.89–8.93)		3,510 (1,420, 7,860)		20.7 (7.64, 49)	
100 mg QD	1.48 (0.623–3.28)	36.5	2,910 (1,220, 6,270)	83	22.2 (8.47, 50.3)	107
200 mg QD	2.97 (1.21–6.68)	73.1	5,850 (2,380, 13,000)	167	44.3 (16.6, 102)	214
200 mg QD × 56 days, then 100 mg QD	2.45 (1.08–5.26)	60.2	3,990 (1,600, 8,980)	114	23.3 (8.48, 57.4)	113
Nonblack patients						
Labeled regimen: 400 mg QD × 14 days, then 200 mg thrice wkly	4.3 (2.01–9.11)		4,350 (1,880, 9,850)		27 (10.6, 64.3)	
100 mg QD	1.73 (0.753–3.87)	40.3	3,600 (1,580, 7,800)	82.6	28.6 (11.6, 64.2)	106
200 mg QD	3.45 (1.58–7.46)	80.3	7,220 (3,240, 15,300)	166	57.4 (23.9, 126)	212
200 mg QD × 56 days, then 100 mg QD	2.75 (1.24–5.98)	64.1	5,130 (2,190, 11,400)	118	32.2 (12.6, 76.4)	119

a All values rounded to 3 significant figures. *C_max_* taken from the day with highest exposure for the respective regimens. Cumulative (cuml.) AUC calculated at 24 h post-final dose (after 24 weeks of dosing). AUC at end of treatment calculated from the average daily AUC over the final week of dosing (AUCwk/7).

**FIG 3:**
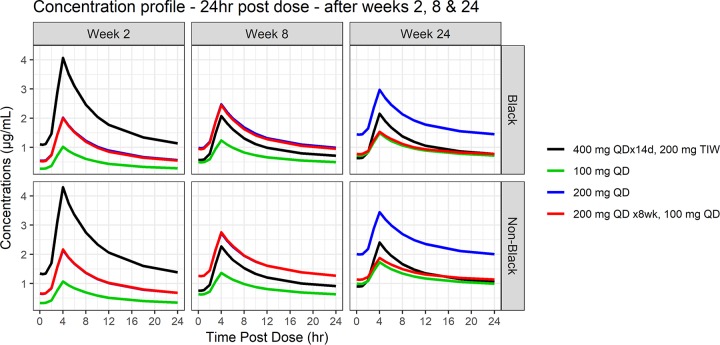
Simulated bedaquiline concentrations over the 24 h postdose after 2, 8, and 24 weeks of dosing. Shown separately for black and nonblack patients.

Fig. 3 compares the median bedaquiline pharmacokinetic profiles, separately for black and nonblack patients, over a 24-h period following 2, 8, and 24 weeks of dosing, thus allowing comparison of the profiles on the day that yields the highest exposures over the 6 months of dosing for each of the regimens of interest. For the labeled regimen, the highest exposure occurs on day 14 following the final 400 mg loading dose. For the 100 or 200 mg QD regimens, this occurs with the last dose at the end of treatment (i.e., the end of month 6). The highest exposures with the regimen of 200 mg QD for 8 weeks followed by 100 mg QD occurs at the end of week 8. All regimens of interest had lower *C_max_* than the labeled regimen on the day of highest exposures (with the exception of *C_max_* of M2 for nonblack subjects per the Svensson model; see the Supplemental Material).

**FIG 4:**
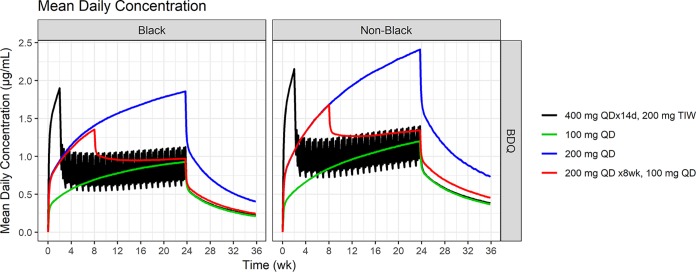
Simulated median of mean daily bedaquiline concentrations over time for selected dosing regimens. Shown separately for black and nonblack patients.

**FIG 5:**
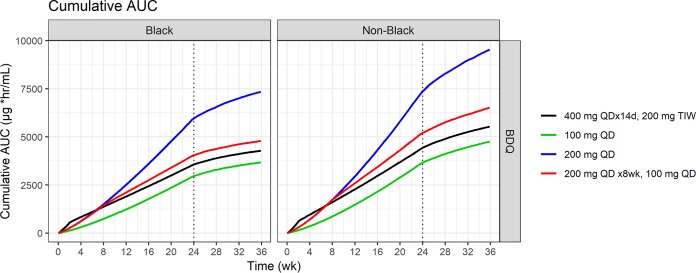
Simulated median cumulative bedaquiline exposure (AUC) over time for selected dosing. Shown separately for black and nonblack patients.

Fig. 4 and 5 depict the simulated median time course of average daily bedaquiline exposure and cumulative exposure (AUC), respectively, separately for black and nonblack patients.

Compared to the labeled regimen, 200 mg QD for 2 months followed by 100 mg QD provides comparable exposures (<20% increase in cumulative or daily exposure at the end of treatment for both bedaquiline and M2).

Simulations based on the Svensson model (Fig. S3 to S5 and Table S1 in the Supplemental Material) provide similar conclusions based on bedaquiline exposures and also based on metabolite M2 exposures, with the exception that nonblack patients were predicted to have slightly higher M2 *C_max_* after 8 weeks on 200 mg QD than on the labeled regimen.

### Conclusions.

The published bedaquiline population pharmacokinetic models (5, 6) predicted a test data set from the NC-005 clinical trial, providing external validation of the model and confidence in applying the model to explore alternative bedaquiline regimens.

Bedaquiline administered 200 mg QD for 2 months followed by 100 mg QD for the remainder of a 6-month regimen appears rational when compared to exposures associated with the labeled regimen (400 mg QD for 14 days followed by 200 mg TIW).

With the labeled regimen, an exposure/response relationship was observed (and modeled) over time for the proportion of patients with sputum culture conversion (8). However, the relatively small difference in median exposure over time between the labeled and proposed regimens (especially compared with between-subject variation) would not likely express as meaningful differences in response. Regarding safety, M2 exposures are correlated with QT prolongation (2). The proposed QD regimen is expected to yield similar pharmacokinetic exposures compared to the labeled regimen. Given the similarities in exposure, the safety and efficacy associated with the new QD regimen are expected to be similar to the labeled dose. The higher simulated *C_max_* of M2 for the new regimen for nonblack subjects was only 4% higher and is thus unlikely to compromise safety. On the other hand, the simpler QD regimen may be expected to benefit from increased adherence. Ultimately, however, the test of these expectations will come from the clinic. Based in part on the work presented herein, the safety and efficacy of this proposed daily regimen are currently being evaluated in the ZeNix (3) and SimpliciTB (4) clinical trials.

## Supplementary Material

Supplemental file 1
