# Supplementary material for: Daily Dosing for Bedaquiline in Patients with Tuberculosis
Source: Antimicrob Agents Chemother. 2019 Oct 22;63(11):e00463-19. doi: 10.1128/AAC.00463-19 (PMC6811417; doi:10.1128/AAC.00463-19)
Supplement: Supplemental file 1 [file AAC.00463-19-s0001.pdf]

Daily Dosing for Bedaquiline in Patients with Tuberculosis

Supplemental Materials

David H Salinger[1], Jerry R Nedelman[2], Carl Mendel[2], Melvin Spigelman[2], David J Hermann[3]

[1]Certara, Inc., under contract with the Bill and Melinda Gates Foundation, [2]TB Alliance, [3]Bill and Melinda Gates Foundation, Corresponding Author

Figure S1: Observed and simulated bedaquiline and M2 trough concentrations over time by regimen and patient type. Visual predictive check of simulations and observed data from NC-005 (Svensson model).

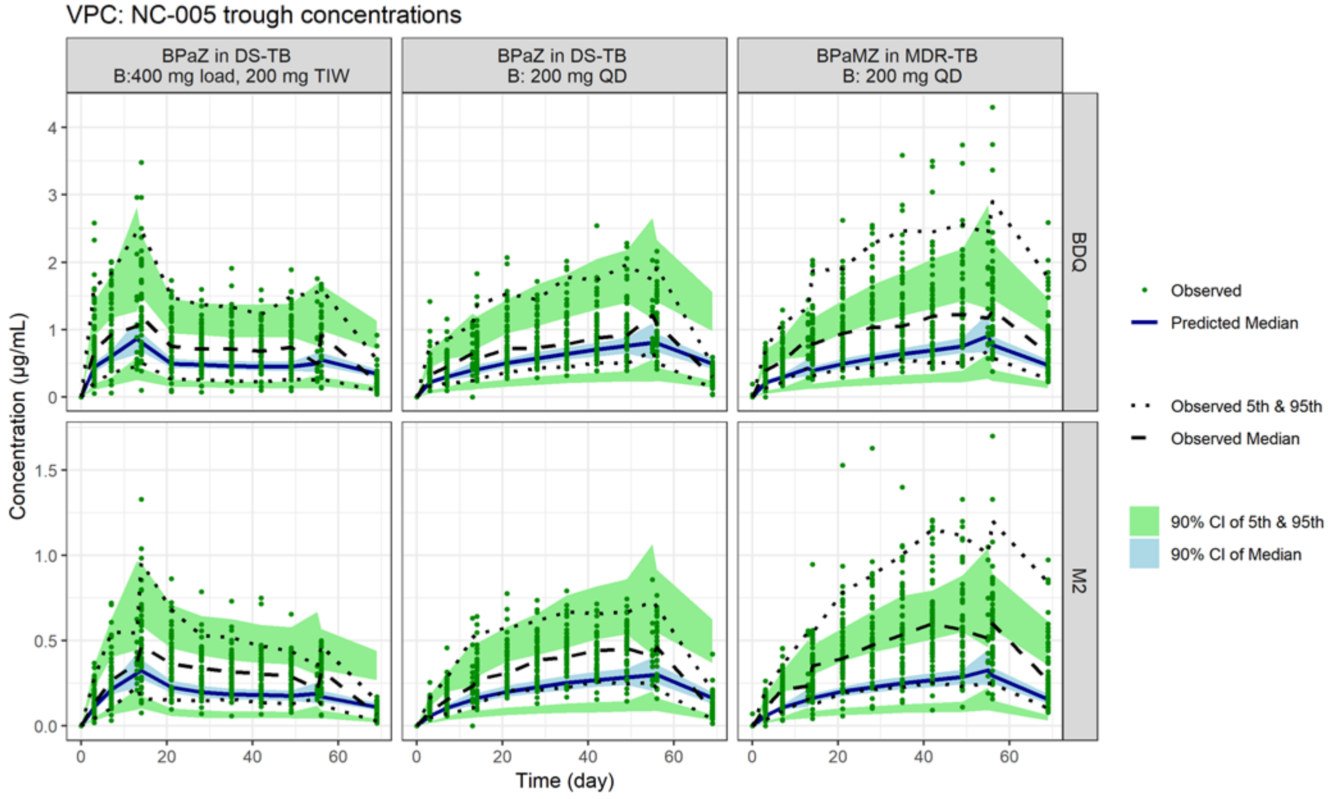

Note: Median and 5th and 95th percentiles of observed bedaquiline and M2 trough concentrations and predicted 90%CI of median and 5th and 95th percentiles of predicted trough concentrations.

B: bedaquiline; M: moxifloxacin; Pa: pretomanid; Z: pyrazinamide; CI: confidence interval

Figure S2a: Observed and simulated bedaquiline and M2 concentrations post final dose (day 14) over time by regimen and patient type. Visual predictive check of simulations and observed data from NC-005 (Svensson model).

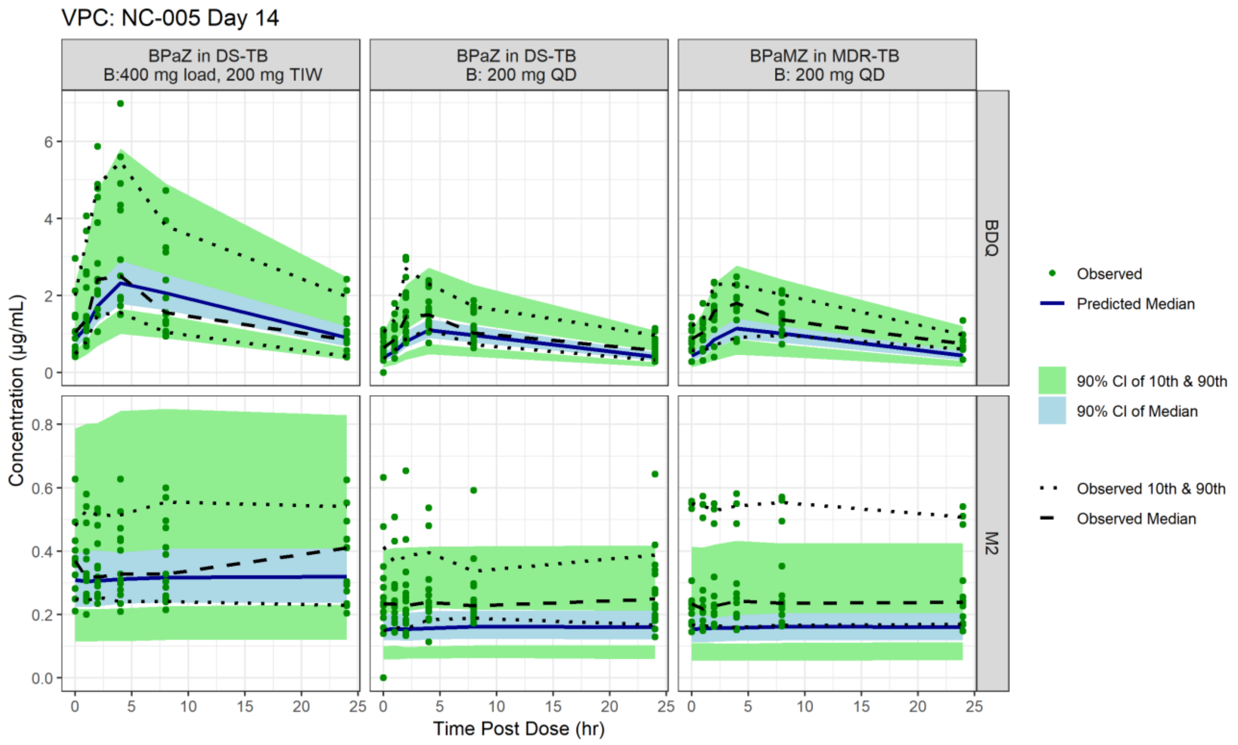

Note: Median and 10th and 90th percentiles of observed bedaquiline and M2 concentrations and predicted 90%CI of median and 10th and 90th percentiles of predicted concentrations post dose on day 14.

80% prediction interval (10th and 90th percentiles) is used due to the small number of subjects per arm (15) included in the substudy

B: bedaqquline; M: moxifloxacin; Pa: pretomanid; Z: pyrazinamide; CI: confidence interval

Figure S2b: Observed and simulated bedaquiline and M2 concentrations post final dose (day 56) over time by regimen and patient type. Visual predictive check of simulations and observed data from NC-005 (Svensson model).

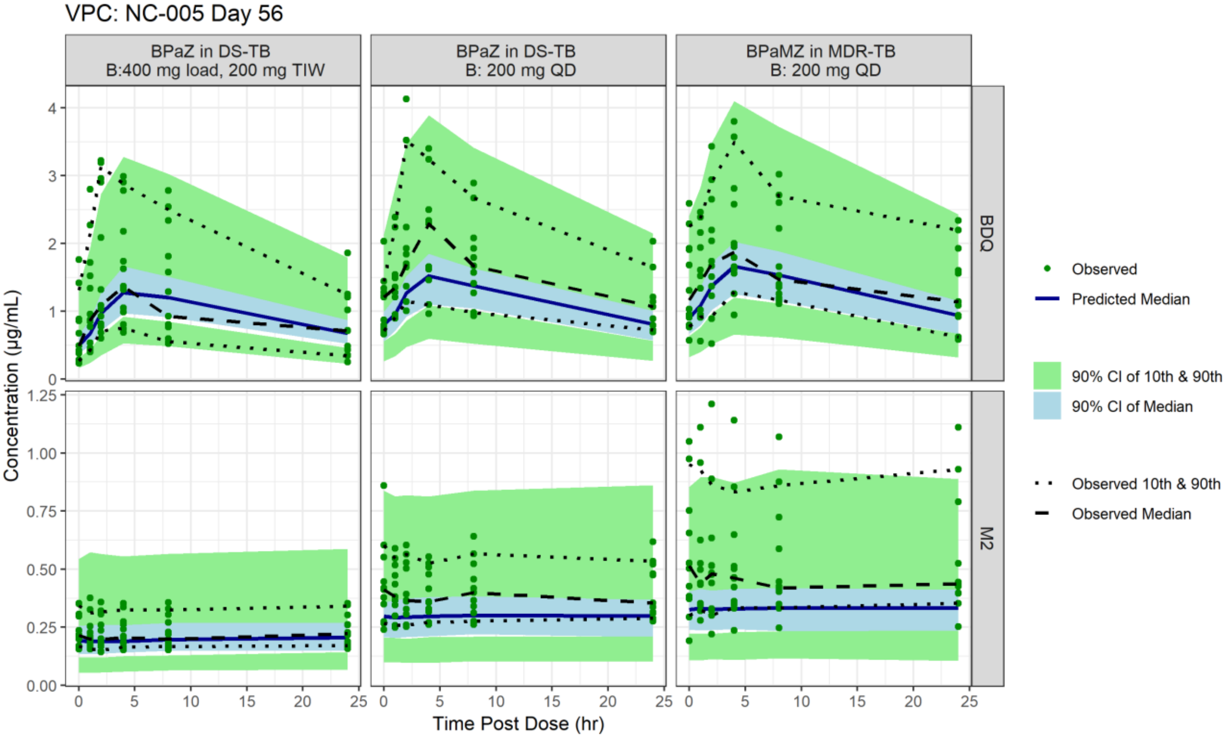

Note: Median and 10th and 90th percentiles of observed bedaquiline and M2 concentrations and predicted 90%CI of median and 10th and 90th percentiles of predicted concentrations post dose on day 56.

80% prediction interval (10th and 90th percentiles) is used due to the small number of subjects per arm (15) included in the substudy

B: bedaqline; M: moxifloxacin; Pa: pretomanid; Z: pyrazinamide; CI: confidence interval

Figure S3a: Simulated bedaquiline and M2 concentrations over the 24 hours post dose after 2, 8, and 24 weeks of dosing – for black patients (Svensson model)

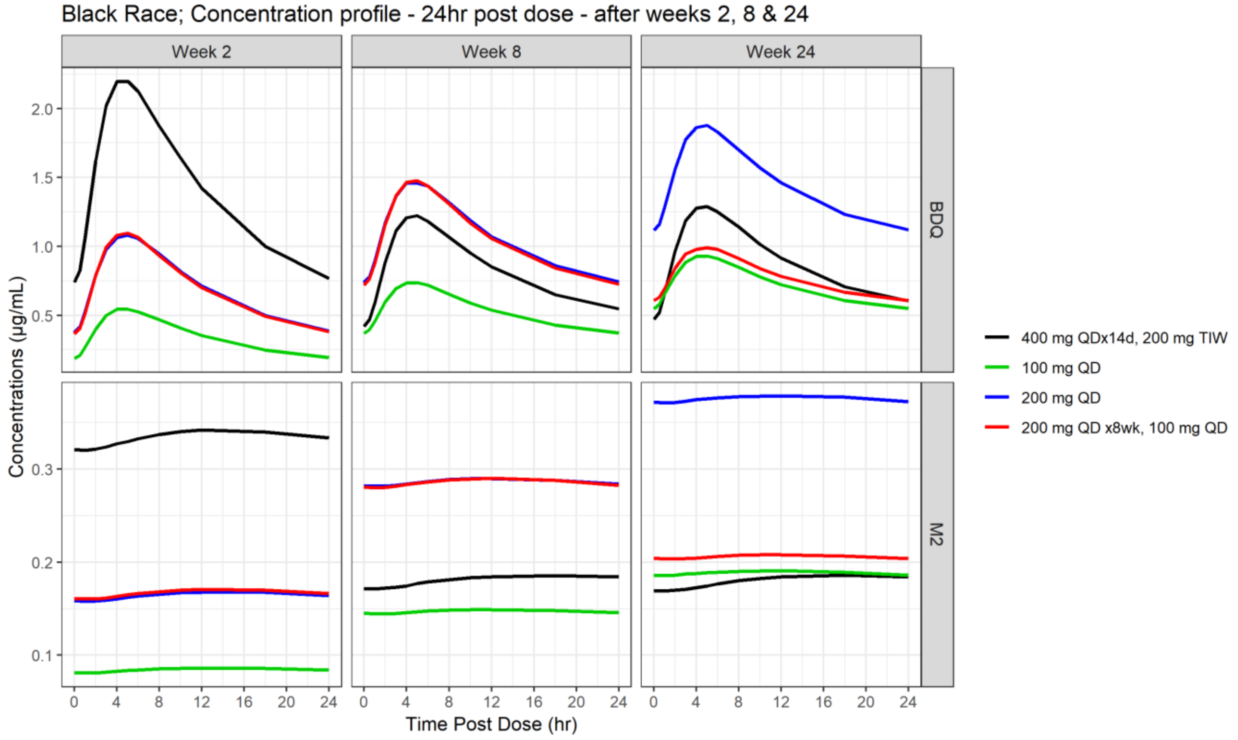

Figure S3b: Simulated bedaquiline and M2 concentrations over the 24 hours post dose after 2, 8, and 24 weeks of dosing – for non-black patients (Svensson model)

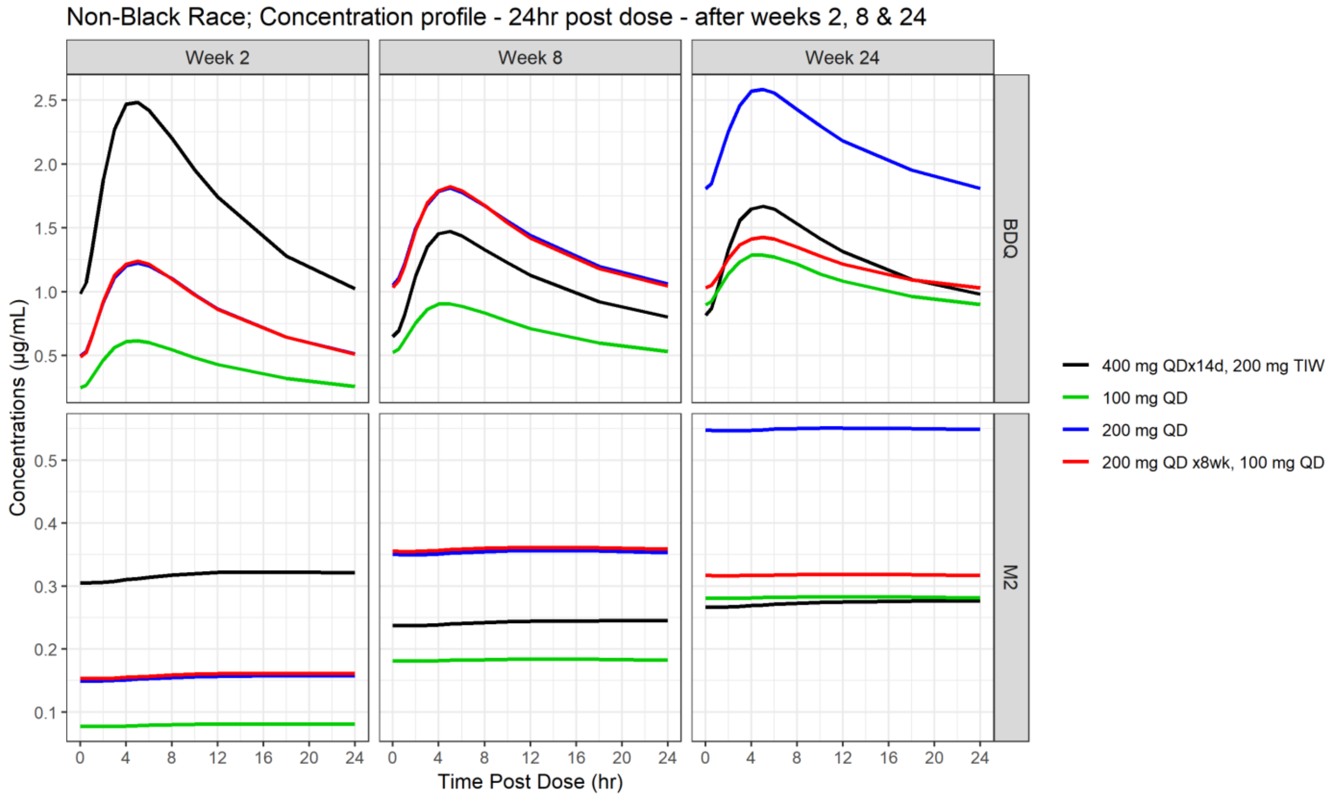

Figure S4: Simulated median of mean daily bedaquiline and M2 concentrations over time for selected dosing regimens – separately for black and non-black patients (Svensson model).

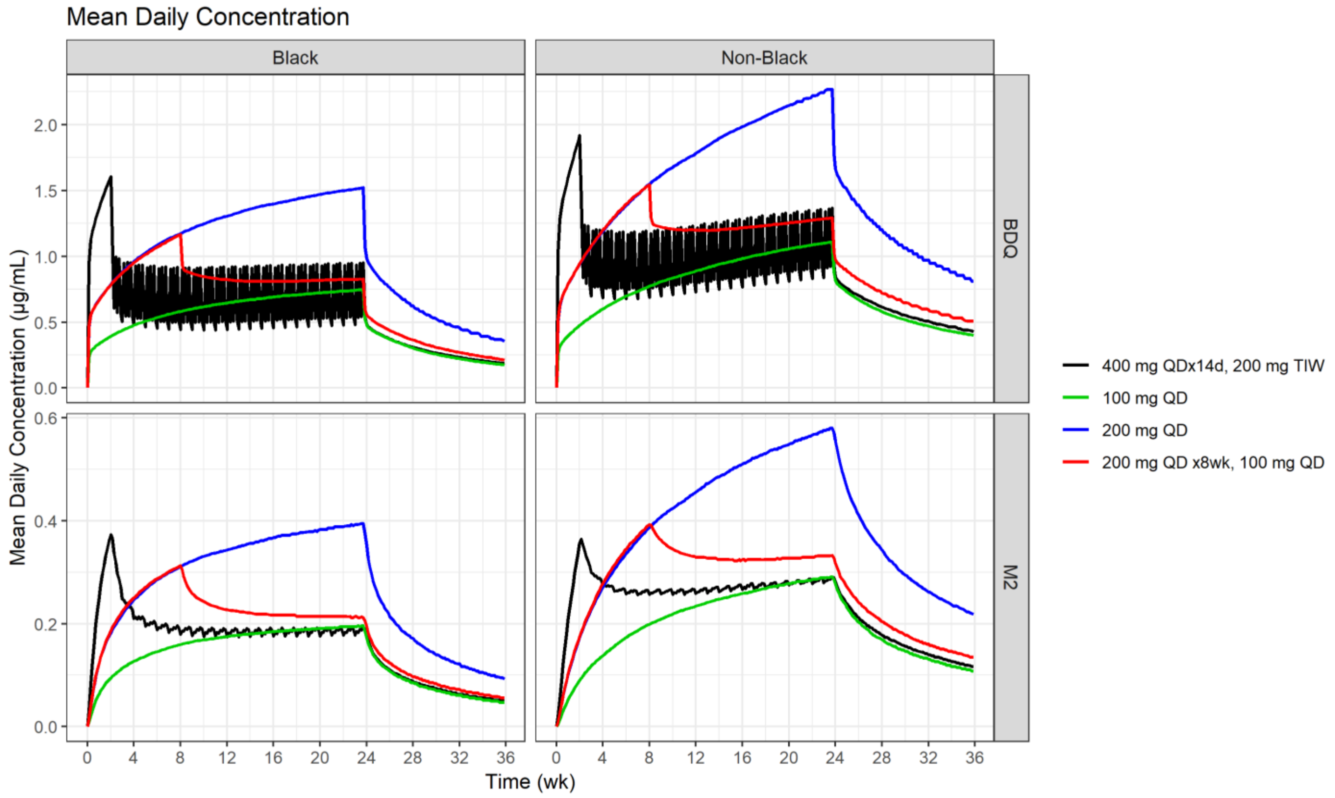

Figure S5: Simulated median cumulative bedaquiline and M2 exposure (AUC) over time for selected dosing – separately for black and non-black patients (Svensson model).

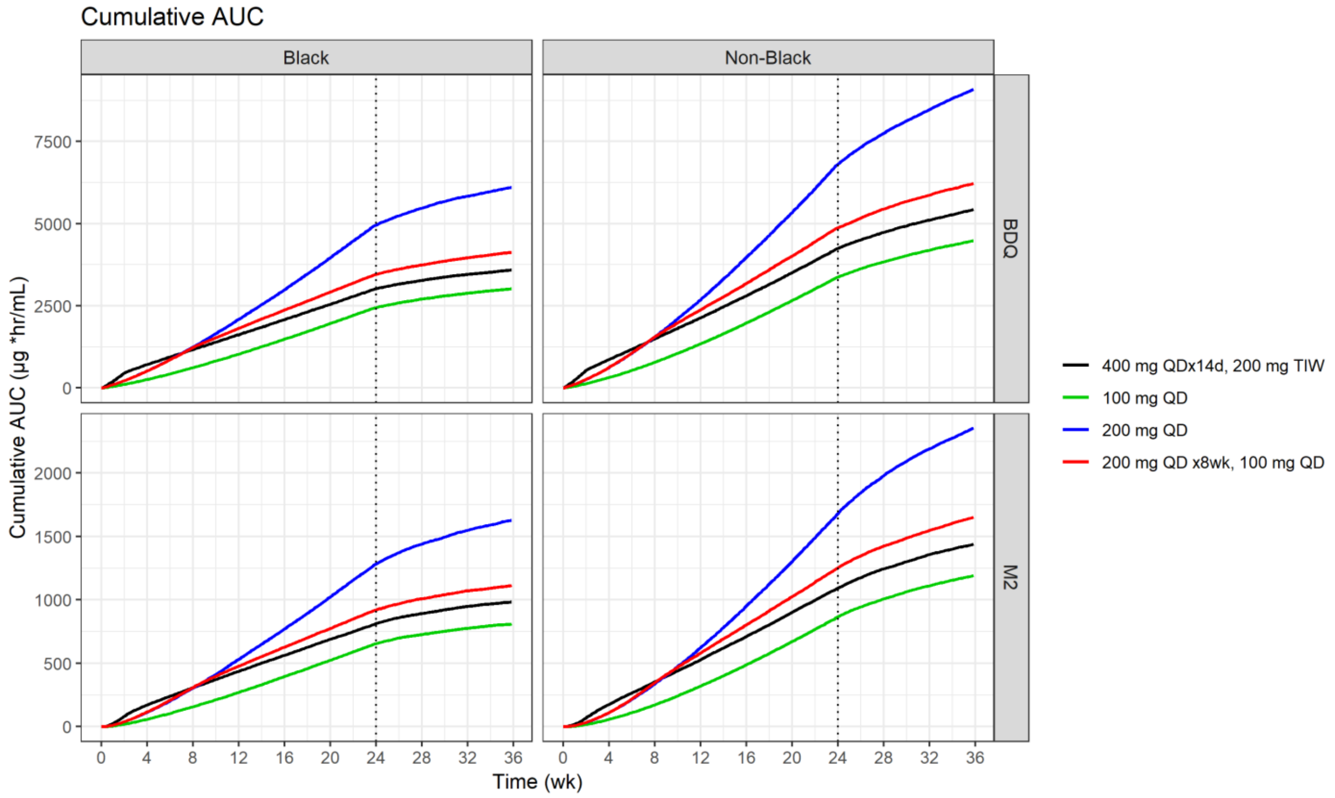

Table S1a: Summary of predicted median (5th, 95th percentiles) bedaquiline exposure metrics by regimen and race

	Regimen	Cmax (µg/mL)	Cmax % of Standard Regimen	Cum AUC at End of Treatment (µg*hr/ml)	Cum AUC % of Standard Regimen	AUC at End of Treatment (µg*hr/ml)	AUC % of Standard Regimen
Black	Labeled Regimen: 400mg QD X 14 Days then 200mg Thrice Weekly	2.31 (1.08, 4.84)	--	16.9 (7.63, 37)	--	2980 (1400, 6270)	--
	100mg QD	0.963 (0.452, 1.96)	41.8%	17.9 (8.08, 38.7)	106%	2410 (1150, 4920)	80.8%
	200mg QD	1.92 (0.882, 3.88)	83.1%	36.3 (16.5, 77.1)	215%	4880 (2290, 10100)	164%
	200mg QD X 56 Days then 100mg QD	1.53 (0.761, 3.01)	66.3%	19.8 (8.83, 44)	117%	3410 (1600, 7230)	114%

Non-black	Standard Regimen:<br>400mg QD X 14 Days then 200mg Thrice Weekly	2.58<br>(1.22, 5.4)	--	26.3<br>(12.7, 55)	--	4170<br>(2050, 8280)	--
	100mg QD	1.32<br>(0.644, 2.61)	51%	26.4<br>(13, 53.5)	100%	3310<br>(1690, 6550)	79.3%
	200mg QD	2.62<br>(1.25, 5.16)	102%	54.1<br>(25.7, 109)	206%	6660<br>(3310, 13200)	160%
	200mg QD X 56 Days then 100mg QD	1.87<br>(0.964, 3.6)	72.5%	30.9<br>(14.4, 63.8)	117%	4800<br>(2350, 9570)	115%

Note: All values rounded to 3 significant figures. Cmax taken from the day with highest exposure for the respective regimens. Cumulative AUC calculated at 24h post the final dose (after 24 weeks dosing). AUC at End of Treatment calculated from the average daily AUC over the final week of dosing (AUCwk/7)

Table S1b: Summary of predicted median (5th, 95th percentiles) M2 exposure metrics by regimen and race

	Regimen	Cmax (µg/mL)	Cmax % of Standard Regimen	Cum AUC at End of Treatment (µg*hr/ml)	Cum AUC % of Standard Regimen	AUC at End of Treatment (µg*hr/ml)	AUC % of Standard Regimen
Black	Labeled Regimen: 400mg QD X 14 Days then 200mg Thrice Weekly	0.347 (0.151, 0.747)	--	4.47 (1.74, 10.8)	--	799 (322, 1920)	--
	100mg QD	0.192 (0.0761, 0.454)	55.2%	4.67 (1.84, 11.8)	104%	642 (250, 1540)	80.4%
	200mg QD	0.38 (0.15, 0.947)	110%	9.42 (3.78, 23.6)	211%	1260 (514, 3070)	157%
	200mg QD X 56 Days then 100mg QD	0.292 (0.118, 0.667)	84%	5.11 (2.01, 13.2)	114%	907 (354, 2210)	114%
Non-Black	Labeled Regimen: 400mg QD X 14 Days then 200mg Thrice Weekly	0.361 (0.16, 0.76)	--	6.86 (2.8, 15.7)	--	1070 (455, 2420)	--
	100mg QD	0.283 (0.115, 0.651)	78.5%	6.94 (2.83, 16.5)	101%	844 (348, 1930)	78.6%
	200mg QD	0.553	153%	13.8	201%	1640	153%

		(0.222, 1.31)		(5.75, 33)		(704, 3930)	
	200mg QD X 56 Days then 100mg QD	0.374<br>(0.159, 0.832)	104%	7.96<br>(3.27, 19.2)	116%	1230<br>(503, 2820)	114%

Note: All values rounded to 3 significant figures. Cmax taken from the day with highest exposure for the respective regimens. Cumulative AUC calculated at 24h post the final dose (after 24 weeks dosing). AUC at End of Treatment calculated from the average daily AUC over the final week of dosing (AUCwk/7)

Nonmem model code for McLeay model

```
$SUBROUTINE ADVAN13 TOL=6
$MODEL          COMP(DEPOT)    ; 1
                COMP(CENTRAL,DEFOBS) ; 2
                COMP(PERIPH1)  ; 3
                COMP(PERIPH2) ; 4
                COMP(PERIPH3) ; 5
                COMP(AUCP)   ; 6

;---PK------------------------------------------
$PK
;; assumptions
MDR = 0  ; DS-TB
SEX= 1   ; Male

KA = THETA(1)
 ; Total dose split between cmt 1 & 2 with fraction FR1, FR2
LGTFR1 = LOG(THETA(2))/(1-THETA(2))
```

```
FR1 = ( EXP(LGTFR1 +ETA(1))/(1+EXP(LGTFR1 +ETA(1))) )
FR2 = 1-FR1
CL = THETA(3)*(1+ BLACK *THETA(15)) *(1 +(1-MDR)*THETA(17))  *EXP(ETA(3)) ; CL inc for black (vs white) and for HV/DS (vs MDR)
VC = THETA(4)*(1+ (1-SEX) *THETA(18)) *EXP(ETA(4))                    ;   VC dec for F vs M
QP1 = THETA(5)
VP1 = THETA(6)
QP2 = THETA(7)
VP2 = THETA(8)
QP3 = THETA(9)
VP3 = THETA(10)
ALAG1 = THETA(11)
ALAG2 = ALAG1 + THETA(12)
D1 = THETA(13)
D2 = THETA(14)

; F is increased for HV/DS (vs MDR).
FTOTAL = THETA(16)**(1-MDR)  *EXP(ETA(2));  F =1 for MDR; =1.51 for DS&HV
F1 = FR1 *FTOTAL
F2 = FR2 *FTOTAL

KEL = CL/VC
K23 =QP1/VC
K32 =QP1/VP1
K24 =QP2/VC
K42 =QP2/VP2
K25 =QP3/VC
K52 =QP3/VP3
```

```
;--- PK diff equations [PK=1:3] -----------------------------------------------
$DES
DADT(1) = -KA*A(1); Absorp (large)
DADT(2) =  KA*A(1) -KEL*A(2)  +K32*A(3) +K42*A(4) +K52*A(5) -K23*A(2) -K24*A(2) -K25*A(2)
DADT(3) = K23*A(2) -K32*A(3)
DADT(4) = K24*A(2) -K42*A(4)
DADT(5) = K25*A(2) -K52*A(5)
; AUCs
DADT(6) = A(2)/VC


$ERROR
C2 = A(2)/VC
IPRED =  A(2)/VC
AUC = A(6)


Y=IPRED +IPRED*ERR(1)

$THETA  ;;; units:  hours, liters, L/hr .
1000 ;    1 KA (large=instantaneous)
0.585  ;  2 F1
2.78  ;   3 CL
164 ;     4 VC
11.8 ;    5 QP1
178  ;    6 VP1
8.03   ;  7 QP2
3010   ;  8 VP2
3.58   ;  9 QP3
7350   ;  10 VP3
0.917 ;   11 ALAG1
1.48 ;    12 ALAG2
2.22 ;    13 D1
1.48 ;    14 D2
```

```
0.52 ;     15 BLACK   52% inc in CL for black
1.51 ;     16 increased F for HV/DS  vs MDR
0.375;     17 inc. CL for HV/DS  vs MDR
-.157;     18 dec. VC for Female vs Male

; $OMEGA
; 1.28  FIX   ; 1 113 %CV on FR1 (logit constrained)
; 0.157 FIX   ; 2  39.6 %CV on F
;
; $OMEGA BLOCK(2)
; 0.254                 ; 3 50.4 %CV on CL,
; 0.08  0.153           ; 4  corr=0.407, 39.1 %CV on VC; 0  FIX  ; 1

$SIGMA
0 FIX   ;;0.042 ;   20.6% CV

$SIMULATION ONLYSIM (8675309)
```
